# Conference Report: DATA ADMINISTRATION MANAGEMENT ASSOCIATION SYMPOSIUM Gaithersburg, MD May 12–13, 1992

**DOI:** 10.6028/jres.097.028

**Published:** 1992

**Authors:** Judith Newton

**Affiliations:** Information Systems Engineering, Computer Systems Laboratory, National Institute of Standards and Technology, Gaithersburg, MD 20899

## 1. Introduction

Along with capital and human resources, an organization’s data represents one of its fundamental assets. Data administration (DA) attempts the effective planning, organization, and management of an enterprise’s data resource, with the intention of empowering the organization to achieve its mission and goals. Very often, however, this endeavor is received with skepticism and limited support by those who could most benefit from data administration practices and products.

The Data Administration Management Association (DAMA) is the professional organization for data administrators. An international board oversees a loose federation of local chapters in the United States, Canada, and Australia. The National Capital Region Chapter (NCR DAMA) has monthly meetings from September through April, as well as a Symposium in May.

NCR DAMA held its fifth annual Symposium at NIST on May 12–13, 1992. The theme this year was *Data Administration–A Value Added Service.* Attended by 275 Federal and private industry data administrators, the Symposium was cosponsored by NIST and NCR DAMA.

It emphasized the practices, technologies, activities, initiatives and ideas that deliver clearly visible value to the users, or “customers” of data administration. In addition to presentations by nationally recognized experts and practitioners, it included workshops, panel discussions, and a tutorial. Topics ranged from the keynote speech on the politics of successful data sharing to the latest implementation of the Information Resource Dictionary System (IRDS) standard.

## 2. Speakers

The keynote speaker was Barbara von Halle, a principal at Spectrum Technology Group, Inc. She described how enterprise-wide data sharing can be a vision and eventually a reality. Because it is a significant cultural change, it can happen in an orderly manner, chaotically, or not at all. She presented practical tips and techniques that have been successful in various organizations, with insight into reasons for failures. There is a delicate balance of short term and long term deliverables and realistic expectations.

Charles Bachman of Bachman Information Systems, Inc. described the IBM AD/Cycle Enterprise Model as “the foundation of the most important technical advance in application development since the advent of COBOL.” The key challenges it must meet are: first, it must be acceptable to the data processing community as the next step in the definition of application systems. Second, it must serve as the semantic target for reverse engineering existing applications. Third, it must be understandable by knowledgeable functional managers. Fourth, it must represent a complete and executable functional specification, subject to logic level debugging. Finally, it must serve as the basis for forward engineering the business requirements to a number of diverse information technology platforms.

Sooner or later, claims Cynthia L. Walker of Software Solutions, Inc., all data administrators are faced with the challenge of justifying their existence by demonstrating true return on investment (ROI). Their challenge is to attempt not only to prove that their organization is performing better and saving time and money as a result of data administration, but also to identify just how much better performance is and exactly how much time and money the organization is saving. The solution has five points of attack: be creative; emphasize tangible, measurable benefits; select an ROI approach that matches your organization’s development stage; avoid initiation stage “quicksand”; and reduce investment (costs).

Andrea Tyndall Norris presented an overview of information architecture activities at NASA, where she is data administrator for the Automated Information Management (AIM) program.

The business view of data management was discussed by Dan Appleton of D. Appleton Co., Inc. During the past 20 years, business management has been steadily reengineering business into more adaptive structures. Adaptive organizations cannot be effective without order of magnitude increases in information quality. The key to achieving these increases lies with data management, particularly with its ability to effectively develop, manage, and employ business rules.

Anthony J. Winkler of ASYSA, Inc. asserted that data administration is a function that exists in many different enterprises, but remains ill-defined as to purpose. It has a critical role in supporting the cost-effective acquisition and operation of information systems, but its role is not always well understood. A model for developing a data administration activity must be driven by a stated set of objectives, engineering the activity to achieve its proper role.

“Winning friends and influencing people” through data administration was the topic of Cathy Hirsh’s presentation. The AMS principal explored how DA programs have been successful in the past and what organizations can do to ensure they remain so in the future. Historically, these have involved three approaches: the “control,” the “you better not..” and the “try it, you’ll like it” approach. Four of the challenges in today’s environment are: the need for more integrated data; the need for using new systems and technology without discarding the old; poor quality data; and faster and better quality development of information technology capabilities.

## 3. Tutorial

Sandra K. Perez of Concept Technology, Inc., presented a tutorial session on “Adding Value with Conceptual Modeling.” She described the method of conceptual modeling, compared and contrasted its expressiveness with different modeling paradigms, and demonstrated its use. These techniques and methods can add value to data administration and effect a positive flow for the effective management of enterprise information resources.

## 4. Panels

The following panel discussions were presented:

- “CASE Studies,” Margaret H. Law, NIST, moderator;
- “Federal Experiences in Data Adminstration,” Bruce K. Rosen, NIST, moderator;
- “DA and the System Life Cycle or DA Versus the System Life Cycle,” Dennis Berg, AMS, moderator;
- “Data Administration Practitioners,” Joseph H. Oates, Life Cycle Technologies, moderator;
- “IRDS Implementation,” Alan Goldfine, NIST, moderator.

## 5. Standards and Procedures Workshops

The Working Group on Standards and Procedures has been meeting monthly during the past year. It has produced a draft Model Data Administration Standards Manual, which was distributed for comment. When finalized, the document will be circulated as a guide to the administration of information standards and include procedures for standards implementation. The group plans to resume monthly meetings in the fall.

Four workshops at the Symposium presented aspects of the working group’s results.

- Data Standardization, Dan Wahl, Vector Research, Inc., and Judith Newton, NIST, moderators;
- Automated Tools, Joan Sziede, U. S. Geological Survey, moderator;
- Data Administration and Security, David Stowell, Stowell Associates, moderator;
- Repository Management, Bill Brimberry, National Park Service, moderator.

## 6. Proceedings

The proceedings of this Symposium were distributed at the event. The Proceedings of the First,^1^ Second,^2^ and Fourth^3^ Annual DAMA Symposia were published by NIST and copies are still available. The Sixth Annual Symposium will be held at NIST May 11–12, 1993.

## Footnotes

1 Judith J. Newton and Frankie E. Spiclman, eds., Data Administration: Management and Practice, Proceedings of the First DAMA Symposium, NIST Special Publication 500-159, National Institute of Standards and Technology, Gaithersburg, MD, October, 1988.

2 Judith J. Newton and Frankie E. Spielman, eds., Data Administration: Standards and Techniques, Proceedings of the Second Annual DAMA Symposium, NISTIR 90-4292, National Institute of Standards and Technology, Gaithersburg, MD, April 1990.

3 Judith J. Newton, Mary Lou Melley, and Holly Higgins, eds., Managing Data: From Vision to Reality, Proceedings of the Fourth Annual DAMA Symposium, NISTIR 4843, National Institute of Standards and Technology, Gaithersburg, MD, May 1992.

